# Assessment of the Efficacy of Physical Activity Level and Lifestyle Behavior Interventions Applying Social Cognitive Theory for Overweight and Obese Girl Adolescents

**Published:** 2018-04-07

**Authors:** Mohammad Bagherniya, Firoozeh Mostafavi Darani, Manoj Sharma, Mohammad Reza Maracy, Ramesh Allipour Birgani, Golnaz Ranjbar, Ali Taghipour, Mohammad Safarian, Seyed Ali Keshavarz

**Affiliations:** ^1^Student Research Committee, Department of Nutrition, Faculty of Medicine, Mashhad University of Medical Sciences, Mashhad, Iran; ^2^Department of Clinical Nutrition, School of Nutritional Sciences and Dietetics, Tehran University of Medical Sciences, Tehran, Iran; ^3^Department of Health Education and Promotion, School of Health, Isfahan University of Medical Sciences, Isfahan, Iran; ^4^Department of Behavioral and Environmental Health, Jackson State University, Jackson, MS, United States; ^5^Department of Epidemiology & Biostatistics, School of Health, Isfahan University of Medical Sciences, Isfahan, Iran; ^6^Department of Community Nutrition, School of Nutritional Sciences and Dietetics, Tehran University of Medical Sciences, Tehran, Iran; ^7^Department of Nutrition, Faculty of Medicine, Mashhad University of Medical Sciences, Mashhad, Iran; ^8^Health Sciences Research Center, Cancer Research Center, Department of Biostatistics and Epidemiology, School of Health, Mashhad University of Medical Sciences, Mashhad, Iran; ^9^Metabolic Syndrome Research Center, School of Medicine, Mashhad University of Medical Sciences, Mashhad, Iran

**Keywords:** Adolescent, Obesity, Physical activity, Schools

## Abstract

**Background:** Childhood obesity has become a global epidemic and physical inactivity and considered asone of the most important contributing factors. We aimed to evaluate a school-based physical activityintervention using social cognitive theory (SCT) to increase physical activity behavior in order to preventobesity among overweight and obese adolescent girls.

**Study design:** Randomized controlled trial study.

**Methods:** A seven-month randomized controlled trial based on SCT was implemented with 172overweight and obese girl students (87 in intervention and 85 in control group), with the presence of theirparents and teachers. Activities of the trial included: Sports workshops, physical-activity consulting privatesessions, free practical and competitive sports sessions, family exercise sessions, text messages, andnewsletters. Body Mass Index (BMI) and Waist Circumference (WC) were measured and questionnairesabout duration of physical activity, duration of screen time (watching television and playing computergames) and psychological variables regarding the SCT constructs were obtained.

**Results:** Duration of physical activity (in minutes) and most of psychological variables (self-efficacy, socialsupport, and intention) significantly increased at post-intervention, while hours of watching television andplaying computer were significantly decreased (*P*<0.001). The subjects’ mean BMI and WC reduced inthe intervention group from 29.47 (4.05) kg/m2 to 28.5 (4.35) kg/m^2^ and 89.65 (8.15) cm to 86.54 (9.76)cm, although they were not statistically significant compared with the control group (*P*=0.127 and P*P*=0.504,respectively).

**Conclusions:** School-based intervention using SCT led to an increase in the duration of physical activityand reduction in the duration of screen time in overweight and obese adolescent girls.

## Introduction

The World Health Organization has estimated that physical inactivity, overweight, and obesity caused 1.9 and 2.6 million deaths throughout the world respectively^1^. Globally, childhood obesity has become one of the most serious public health challenges of the twenty-first century^2^. The prevalence of childhood and adolescent overweight and obesity have doubled in the three decades up to 2012^3^. The prevalence of overweight and obesity among Iranian children and adolescents has been recently estimated at about 5.1% and 10.8%, respectively^4^. Overweight and obesity in children and adolescents is attributed to several long-term physical and psychological adverse consequences^5^. In addition, lifestyle changes during adulthood for treatment of obesity is proven less likely compared to childhood^6^. Thus, it is necessary to prevent and combat childhood obesity and consider it as a main priority for public health policymakers.

Following unfavorable lifestyles that include unhealthy eating habits and physical inactivity are considered as the most important risk factors, which cause childhood obesity^5^. The rate of physical activity decreases sharply during adolescence and happens especially in girls, which often continues into adulthood and causes long-term low physical activity and low fitness levels^7,9^. The level of physical activity in the adolescents is far below recent recommendations in both industrial and developing countries and those girls are less active than boys^10^. About two-thirds of Iranian adolescents are physically inactive^11^. Increased television viewing and use of computer as well as video games and lack of appropriate places in school and community are considered as the most important reasons for being physically inactive among Iranian adolescents^12^.

However, for prevention of obesity among adolescents, few intervention programs have been designed and implemented in developing countries such as Iran, which could explain the challenges of working with adolescents^8^. Furthermore, due to complexity of the problem, there are not enough well-established universal interventions for prevention of childhood and adolescent obesity. The number of intervention studies, particularly theory-based studies, in middle and low-income countries should be increased^3^. Since children spend many hours in school, it has been considered as the ideal place for nutrition and physical activity interventions^5,13^.

Social cognitive theory (SCT) is among the more practical theories especially for school-based programs, which suggests that human behavior is a result of dynamic interaction between personal, behavioral and environmental factors^14,15^.

The aims of this study were to assess the effects of physical activity education intervention using SCT to increase physical activity in order to prevent obesity among overweight and obese adolescent girls and determine the usage of components and constructs of the theory for Iranian adolescents.

## Methods

### Study design

This randomized control trial study was a part of protocol, approved by the Ethics Committee of Tehran University of Medical Sciences. The design, implementation, and reporting of the trial were based on Consolidated Standards of Reporting Trials (CONSORT) guidelines^16^. This trial was registered in Iranian Registry of Clinical Trials (IRCT2013103115211N1).

**Figure 1:**
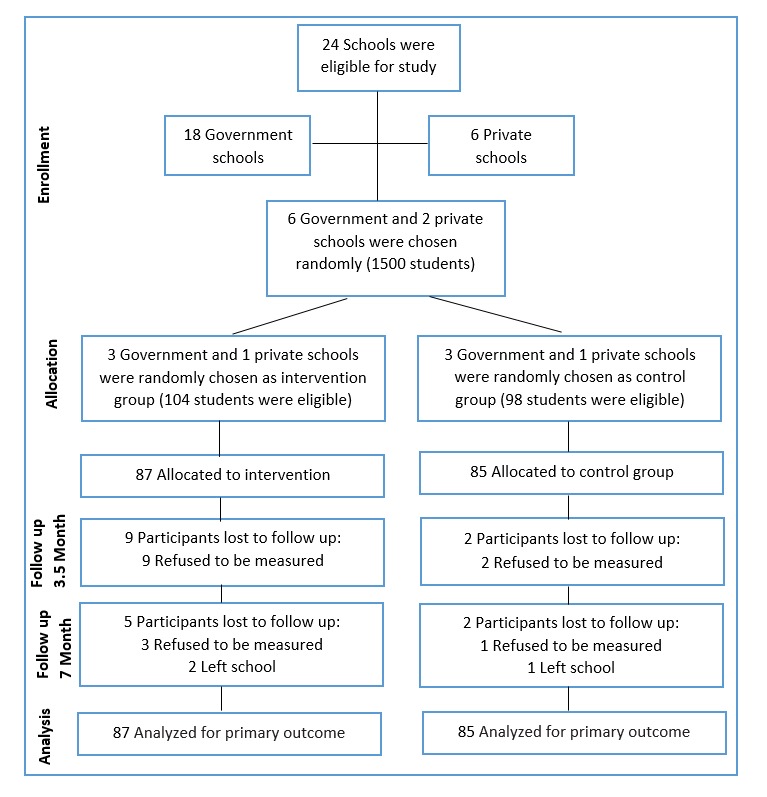
Flowchart of participants throughout the study

The study was conducted among the schools with the same socioeconomic background in Shahinshahr, near the city of Isfahan, centeral Iran in 2014. Among all 24-girl middle schools, eight cases were included in the study; six of these were state-schools (from eighteen state schools) and two were private schools (from six private schools). The schools were randomly selected (Figure 1). The government schools were alphabetically sorted and every two schools were selected as a pair. Then, from each paired school, one was randomly assigned to the intervention group and the other as the control group. This method continued for all of the other paired. One of the research team members randomly selected three schools from government schools and one school from private school as the intervention group. The other schools were thus considered as the control group.

In each school, after checking individual student’s health records, only the overweight and obese adolescents (between 12-16 yr of age) were selected. Both parents and students were asked to fill the consent form before participating in the study. Excluded criteria were diet for losing weight before the study, consuming drugs associated with gaining weight such as nerve drugs and corticosteroids, having any metabolic diseases such as endocrine disorders, cardiovascular diseases, and diabetes. Sample size was calculated based on 80% power, an α level of 0.05, and a potential dropout rate of 10%, 150 participants (ie, 75 participants in each group) would be required to detect 10% differences between-groups. Finally, from about 1500 students who were studying in those eight schools, a total of 202 students from seventh and eighth grades participated in the study. They all met the criteria for being overweight or obese (those whose body mass index (BMI) values are equal or more than 85^th^percentile)^17^.

### Intervention

With the aim of gradual increase in physical activities and decrease in sedentary behaviors, a school-based intervention applying SCT with regard to evidence-based psychological (self-efficacy, outcome expectations, outcome expectancies, intention and perceived barriers) behavioral (knowledge and skills) and environmental (parents, teachers, friends support) components were conducted over seven months (30 wk). The components of the interventions were as follows:

1. *Sports Workshops* :Sport workshops and interactive seminars twice a month for students (14 sessions), once a month for their parents (seven sessions) and four sessions for their teachers in which audiences were taught about the importance of being physically active for well-being, the effect of sports and exercise on health and weight reduction, health disadvantages of high sedentary behaviors like watching television more than two hours a day and having normal weight, the effect of physical activities on reducing chronic diseases with the aims of enhancing their knowledge in sports, changing their attitudes and practices. Social support, outcome expectations, and outcome expectancies were targeted constructs of theory in these sessions, in each session, the instructor focused on positive outcomes of adhering to moderate to intensive physical activities for at least one hour a day and maintaining a healthy body weight and introducing best ways to reduce barriers of being physically active. Parents and teachers were taught to provide rewards for the girls, who increase their physical activities. During the sessions, the students received one educational CD about physical activities, two brochures and one handbook about physical activity to support participants and their parents in their free times especially over the weekends.
2. *Private Consulting Sessions* : Private physical activity consulting sessions were organized on a monthly basis, where seven individual sports counseling (face-to-face) meetings for students accompanied by their parents were held. Targeted constructs of theory were self-efficacy, social support, intention and perceived barrier. At the beginning of each session, students were asked to recall their past 24-h physical activities to obtain type and duration of their physical activities and then notice their problems. In each session, students, their parents, and the researcher discussed the best ways to achieve favorable physical activity levels and decrease sedentary behaviors and set some practical goals. Then the sports expert encouraged students to attain their goals until the next meeting. Furthermore, parents were made responsible to encourage and support the students to achieve their goals. In the next session, if the students could not reach their goals, problem-solving approaches were suggested by the sports expert to find out the reasons of it and help the students to find more effective ways to achieve their goals or set new ones, which were more attainable.
3. *Free practical and fun exercise sessions* : For increasing the duration of physical activities in the subjects, sessions of sport (each session 90 min) were held twice a week (60 sessions in 7 months) delivered by a specialist in a physical education. In these classes, students were taught the correct methods of exercise, which could cause less damage to them. At the beginning of each session, the physical trainer set some achievable goals and asked students to follow their goals to increase their confidence. Increasing self-efficacy and decreasing perceived barriers were also focused by the sports trainer. All classes were fun and enjoyable to encourage subjects to be physically active and there were no competitive games.
4. *Free practical and competitive sports sessions* : Students volunteered to participate in an afterschool competitive gameplay where they could freely register in the nearest gym to their houses for two sessions per week.
5. *Family physical activity sessions* : Three sessions of physical activities included walking, jogging, and mountain climbing. These were held for students and their parents so that parents could encourage their children in sports time and increased their social support.
6. *SMS Text messages for students* : Students received SMS text messages weekly containing the main goals of the program and strategies to overcome barriers to being physically active.
7. *SMS Text messages for parents* : Parents were sent text messages weekly which reminded them to support their children to be physically active.
8. *Parents’ newsletters:* After the end of each month, parents received a newsletter which showed the study progress and motivated them to support their children for achieving their goals. In addition, the newsletters showed the plans of the study in the next month. The newsletters were signed and given back to the study researchers by the parents.
9. *Increasing facilities of physical activity in the schools* : School Principals were asked to provide more sports equipment in the school environment to support students, who intended to be more active in the school.

For ethical purposes, three physical education classes were held for students and two lectures were held for their parents and teachers in the control group and they received one handbook about merits of physical activity. In addition, after the study, students were given 10 tickets for participation in the nearest gyms for free.

### Assessments and measures

*Primary Outcome Measures* : Weight, height and waist circumference (WC) were measured three times, baseline (one week before the intervention), middle (three and half a month after the intervention) and at the end of study (one week after the 30 wk of intervention) by a well-trained nutritionist who was blind to the study allocation. Weight and height were measured in duplicate in subjects without shoes and minimal clothes by Seca digital scale and without shoes by Seca stadiometer and recorded with 0.1 accuracies. WC was obtained in duplicate, from narrowest point between top of the iliac crest and lowest rib margin by flexible tape. For all of these variables, the averages of two measurements were obtained and used for analyses.

*Secondary Outcome Measures* : As previously explained elsewhere^18^, a valid and reliable questionnaire about physical activity SCT constructs was used to assess psychological variables which included self-efficacy, social support, outcome expectations (i.e., perceived benefits) and outcome expectancies (i.e., values placed on benefits), intention (i.e., proximal goals) and perceived barriers. In summary, validated scales were translated into Persian by a bilingual researcher^19,20^. Then, five English fluent sports experts and health education specialists assessed, revised and modified the questionnaire. The questionnaire was translated into English by an Iranian expert in English. After that, in order to test and retest the questionnaire, a pilot study on 20 separated overweight and obese students (more than 10% of study participants) was conducted. Consequently, calculating the Cronbach‘s alpha and test-retest reliability coefficients showed that they were in an acceptable range for all parts of the final questionnaire (i.e. > 0.7). The scale had eight questions about self-efficacy (four choices Likert-type scale), six questions about social support (five choices Likert-type scale), five questions about outcome expectations (six choices Likert-type scale), five questions about outcome expectancies (four choices Likert-type scale), one question about intention (four choices Likert-type scale) and ten questions about perceived barriers (four choices Likert-type scale). Type and time of physical activity and duration of sedentary behaviors such as hours of watching television and hours of playing computer games per day were assessed by physical activity questionnaire^18^. Participants completed the general questionnaire which was about socio-demographic information once at baseline, and they filled other questionnaires at baseline and end of the study.

### Statistics and analysis

All data were analyzed through SPSS ver. 18 (Chicago, IL, USA). The Kolmogorov-Smirnov test was used to evaluate the normal distribution of the variables. Chi-square test was applied to show differences between the educational and occupational status of fathers and mothers. Independent *t* -test was used for showing differences between two groups at baseline and the end of the study and if the distribution of variables was not normal, Mann-Whitney *U* tests were used. Mann-Whitney *U* test was used to assess physical activity differences between groups. Wilcoxon test also was used to evaluate physical activity differences within the groups. For assessing changes in SCT variables and duration of sedentary behaviors (outcomes which measured at baseline and end of the study) we applied General Linear Model Univariate analyses. SCT constructs at the end of study were considered as dependent variables and groups as the fixed factor with controlling baseline values of these variables and potential confounders. For assessing differences in variables measured three times (baseline, middle, and end of the study) and the interaction between intervention group and time of follow up, we used General Linear Model analyses of repeated measures with co-variate adjustments for baseline values and for the potential confounding factors include age, sociodemographic factors, energy and dietary intakes. The between-subjects factor was groups (intervention and control) and the within-subjects factor was BMI, WC, and nutritional behaviors. *P* value less than 0.05 were considered as a statistically significant level.

## Results

A total of 202 students met the inclusion criteria of which 172 completed baseline assessments. The intervention and control groups included 87 and 85 students respectively (Figure 1). Nine subjects of intervention group (10%) quit the study in the middle of study (15 wk after baseline), while 73 participants completed the study in this group (84%). In the control group, 85 participants participated until halfway of the study (98%) and 81 of them finished the study (95%). The mean ages in the intervention and control groups were 13.53 (0.67) yr and 13.35 (0.60) yr, respectively (*P* =0.077). Socio-demographic outcomes of the study participants are shown in Table 1.

**Table 1:** The educational and occupational status of fathers and mothers (n=87 in the intervention group and n=85 in the control group)

**Variables**	**Control group**		**Intervention group**		***P*** **value**
	**Number**	**Percent**	**Number**	**Percent**	
Father’s Job					0.421
Government job	32	37.6	38	43.7	
Private job	53	62.4	49	56.3	
Mother’s Job					0.886
Housekeeper	72	84.7	73	83.9	
Government or private jobs	13	15.3	14	16.1	
Father’s Education					0.994
Under Graduate	70	82.4	72	82.8	
University Degree	15	17.6	15	17.2	
Mother’s Education					0.878
Under Graduate	73	85.9	74	85.1	
University Degree	12	14.1	13	14.9	

The comparison within groups showed that duration of physical activities was significantly (*P* <0.001) increased in the intervention group though no changes were found in the duration of physical activities in the control group (Table 2). Further analysis using Mann-Whitney *U* test showed that the mean differences of physical activities between two groups were statistically significant (*P* <0.001) (data were not shown in Table 2). In addition, most of the psychological variables included in the study such as physical activity self-efficacy, social support and intention also increased in the intervention group, statistically significant compared with the control group. In addition, hours of sedentary behaviors and physical activity perceived barriers were reduced in the intervention group, which were statistically significant in comparison to the control group (*P* <0.001) (Table 2).

**Table 2:** Anthropometric Variables and After Study, Physical Activity and Sedentary Behaviors of the study sample and Social Cognitive Theory Constructs at Baseline and End of Study

**Variables**	**Mean (SD)**		***P*** **-value**
	**Baseline**	**Endpoint**	
Body mass index ^a^			0.127
Intervention	29.2 (3.9)	28.5 (4.3)	
Control	27.2 (2.9)	27.6 (2.9)	
*P* value	0.001	0.156	
Waist circumferences ^b^			0.504
Intervention	89.6 (2.9)	86.5 (9.8)	
Control	84.4 (6.7)	84.9 (6.4)	
*P* value	0.001	0.237	
Minutes of daily physical activity			0.001
Intervention	11.7 (16.3)	41.8 (32.1)	
Control	8.6 (12.6)	9.9 (15.4)	
*P* value	0.159	0.001	
Hours of sedentary behaviors			0.001
Intervention	3.2 (1.3)	2.8 (1.4)	
Control	2.7 (1.4)	2.8 (1.3)	
*P* value	0.017	0.788	
Physical activity self-efficacy			0.001
Intervention	13.5 (3.1)	20.7 (6.4)	
Control	13.6 (2.9)	13.7 (2.7)	
*P* value	0.758	0.001	
Physical activity social support			0.001
Intervention	10.9 (3.2)	11.6 (2.7)	
Control	11.1 (3.3)	11.7 (3.1)	
*P* value	0.739	0.307	
Physical activity outcome expectations			0.292
Intervention	22.5 (5.0)	22.7 (4.3)	
Control	22.8 (3.9)	22.7 (3.9)	
*P* value	0.680	0.898	
Physical activity outcome expectancies			0.145
Intervention	17.5 (2.7)	17.7 (2.4)	
Control	17.7 (2.5)	17.6 (2.4)	
*P* value	0.723	0.815	
Physical activity intention			0.001
Intervention	1.6 0(.8)	2.9 (1.1)	
Control	1.5 (0.7)	1.5 (0.7)	
*P* value	0.701	0.001	
Physical activity perceived barriers			0.001
Intervention	25.1 (2.9)	18.6 (5.8)	
Control	25.3 (2.8)	25.2 (2.7)	
*P* value	0.703	0.001	

^a^ follow-up time=0.185; interaction (time-groups)=0.001 ^b^ follow-up time=0.903; interaction (time-groups)=0.001

At baseline between BMI and WC and sedentary behaviors of two groups significant differences existed (*P* <0.001), but it did not in other variables (Table 2). BMI and WC decreased in the intervention group favorably though they were not statistically significant in comparison to the control group (F_(1,148)_ = 2.3 ; *P* =0.127 and F_(1,148)_ = 0.45 ; *P* =0.504, respectively). The interaction between follow up time and intervention group in BMI and WC indicate that the mean difference between two groups in baseline, middle and end of the study were statistically significant (F_(2,147)_ = 22.5 ; *P* <0.001 and F_(2,147)_ = 15.2 ; *P* <0.001 respectively) (Table 2). At baseline, in the intervention group, seven (8%) of participants were overweight and 80 (92%) of them were obese. In the control group, 18 (21.2%) of individuals were overweight and 67 (78.8%) of subjects were obese. At the end of the study, in the intervention group, 17 (23.29%) of subjects were overweight and 56 (76.71%) were obese. In the control group, 18 (22.22%) of participants were overweight and 61 (77.78%) of them were obese.

## Discussion

The main finding of this study was that after a seven-month intervention based on one of the popular behavioral theories, namely social cognitive theory, physical activities increased and screen time decreased among intervened students. There are very few studies in developing countries to evaluate the efficacy of theory-based physical education intervention on overweight and obese adolescents.

Daily minutes of physical activity significantly increased in the intervention group compared with the control group. Low confidence, fear of being ridiculed by other normal weight peers, lack of facilities, inadequate resources, and unsupportive family were considered as the most important reasons of Iranian overweight and obese adolescents for their physical inactivity^21^. Furthermore, since gameplay may not have enough rewards and too many challenges for overweight and obese adolescents, they often dislike competitive sports^22^. On the other hand, competitive sports can cause more calories expenditure^22^. In our study, for participants in the intervention group, we considered both cooperative practical sports sessions, which were enjoyable and funny as well as competitive sports sessions (gameplay); both options were free to provide opportunity for students to exercise in the way that they liked. Our results confirm the results of previous studies^23,25^ all of which increase the duration of physical activities among children and adolescents using educational interventions significantly.

In line with the results of previous studies^8,26^, results of the current study showed significant reduction of sedentary behaviors such as watching television and playing computer in the intervention group in comparison to the control group. Increased duration of physical activity and improvement in knowledge of students and their families about merits of being physically active and adverse consequences of sedentary behaviors could be the possible reasons for these behavior changes.

BMI and WC decreased in the intervention group though it was not statistically significant compared with the control group. However, these reductions in the BMI and WC had a clinical importance in the intervention group. A significant association was found between rate of increase in both weight and BMI during adolescents and fasting insulin, HDL-C and systolic blood pressure in young adults. In addition, excess weight gain during childhood is a risk factor for cardiovascular disease in adulthood^27^.

School-based physical activity programs for children and adolescents had a little effect on BMI^28^. Physical activity intervention on children with six-month to three years duration did not have a favorable positive effect on BMI although they had other benefits on health^29^. However, the BMI may not be an appropriate measure for showing the impact of interventions to increase physical activity in children^28^, and BMI lacks sensitivity to distinguish between fat and fat-free mass during adolescence^30,31^, which might be a possible explanation for results of these studies.

The strengths of the current study were that it included the unique subjects who all were overweight and obese adolescents and applied one of the most frequently used theories in physical activity interventions, namely SCT, and the intense intervention in students, their parents, and teachers. However, this study had some limitations. First, since random sampling was used for school, not for students, un-matched randomization between intervention and control groups at baseline was seen. Second, self-reported questionnaires were used to obtain all data except for anthropometric variables, which could introduce some measurement bias.

## Conclusions

School-based intervention using SCT improved duration of physical activity and most of the physical activity psychological variables and decreased duration of sedentary behaviors in overweight and obese adolescent girls. In addition, BMI and WC decreased in the intervention group although they were not statistically significant in comparison to the control group. To show the usage, improvement, and extension of this theory in developing countries, more studies, especially longitudinal studies will be needed.

## Acknowledgements

The authors would like to thank all students who participated in this study and all school principals and sub-principals who welcomed us in their schools.

## Conflict of interest statement

The authors declare that there is no conflict of interest.

## Funding

This project was supported and founded by Tehran University of Medical Sciences through grant number 23509.

## Highlights

- Theory-based physical education intervention improved psychological variables regarding social cognitive theory constructs.
- Theory-based intervention can effectively increase physical activity among adolescents.
- Intervention based on social cognitive theory reduced sedentary behaviors among adolescents.
